# Exploration of the core gene signatures and mechanisms between NAFLD and sarcopenia through transcriptomic level

**DOI:** 10.3389/fendo.2023.1140804

**Published:** 2023-03-09

**Authors:** Ziying Xu, Zihui Yu, Shang Li, Ziyan Tian, Jing Yuan, Fuping You

**Affiliations:** ^1^ School of Basic Medical Sciences, Peking University, Beijing, China; ^2^ Department of Bacteriology, Capital Institute of Pediatrics, Beijing, China; ^3^ Department of Orthopedics, General Hospital of Chinese People's Liberation Army of China (PLA), Beijing, China; ^4^ National Clinical Research Center for Orthopedics, Sports Medicine & Rehabilitation, Beijing, China

**Keywords:** NAFLD (non-alcoholic fatty liver disease), sarcopenia, high throughput sequencing, bioinformatics analyses, co-expressed genes

## Abstract

**Introduction:**

The increased prevalence of non-alcoholic fatty liver disease (NAFLD) and sarcopenia among the elderly are facing a significant challenge to the world’s health systems. Our study aims to identify the coexpressed genes in NAFLD and sarcopenia patients.

**Methods:**

We downloaded the transcriptome data of NAFLD tissue from patients, as well as muscle tissues from sarcopenia patients, from the GEO database in order to investigate the shared transcriptional regulation mechanisms between these two diseases. Then, focusing on the genes that were frequently expressed in these diseases, together with GSVA and WGCNA, we utilized a range of analysis methods to identify the main co-expressed genes in both diseases by taking intersections. We investigated these changes after learning that they mostly affected lipid metabolism and oxidative stress injury pathways.

**Results:**

By analyzing these genes and their interactions with transcription factors and proteins, we were able to identify 8 genes that share common patterns. From these 8 genes, we were possible to forecast potential future medicines. Our research raises the possibility of NAFLD and sarcopenia transcriptome regulatory pathways in aging populations.

**Discussion:**

In conclusion, a complete transcription pattern mapping was carried out in order to identify the core genes, underlying biological mechanisms, and possible therapeutic targets that regulate aging in NAFLD and sarcopenia patients. It provides novel insights and proof in favor of decreasing the increased prevalence of sarcopenia in the elderly caused by NAFLD.

## 1 Introduction

Non-alcoholic fatty liver disease (NAFLD) is characterized by the accumulation of lipids in the liver, which may further lead to the deterioration of liver fibrosis (1), cirrhosis and even liver cancer (2). According to statistics, about 25% of the world population is suffered from NAFLD, and patients attacked by NAFLD are becoming younger (3). Therefore, it is imperative to underlying the mechanisms of NAFLD and develop an effective treatment for it. Hepatic steatosis is age-related and associated with metabolic syndromes such as high-fat diet (4), flora disorders(5) and hyperlipidemia, as well as various toxins, drugs, and diseases. The pathogenesis of NAFLD is well established as a two-strike and multiple-strike theory but the molecular mechanism of the occurrence and development of NAFLD remains uncovered. (6). However, other aging diseases interrelated with lipid metabolism and fibrosis can aggravate the exacerbation of NAFLD.

Skeletal muscle accounts for 40% of the body weight, undertake 30% of the basic energy metabolism, and maintains behavioral functions. In adults, muscle loss begins at age 30 and accelerates after age 50. By age 60, muscle loss can reach 30 percent. The degeneration of muscle with the increase of age is defined as sarcopenia, which is accompanied by a series of pathological changes such as decreased muscle mass, fibrosis, and fat infiltration, seriously affecting the functional activities of the elderly and reducing life expectancy(7). The prevalence of sarcopenia in people over 80 years of age is as high as 50% and becoming a novel condition with direct life-threatening within the developing world (8) (9). Multiple factors are responsible for muscle glycolipid-metabolism disorder with aging. Reduced oxidative capacity or physical activity with aging both increases the proportion of lipids in body composition, and causes activation of inflammation and insulin resistance(10, 11). Numerous pro-inflammatory cascades are conjunct within muscle and visceral fat, which approach less muscle mass. Additionally, impaired insulin sensitivity can be further increased by muscle catabolism, resulting in abundant ectopic fat deposition within the muscle. Interstitial fibrosis is the other major histopathological change during the progress of sarcopenia, which contributes to the recession of force generation and enhances muscle stiffness.

Glycolipid-metabolism and fibrogenesis appear to be the intersection joint of NAFLD and sarcopenia. With a high degree of functional sharing, crosstalk and mutual regulation, one’s metabolic disorders can lead to compensatory or even systemic metabolic disorders (12). Studies have shown that sarcopenia is an important indicator of the severity of NAFLD (13). Therefore, investigating the association and verifying the shared pathways between them provides a prospective way of creating novel age-related disease treatment strategies. (14).

Transcriptome analysis can determine and quantify changes in transcription levels in various states(15). A large number of applications in the life sciences have made transcriptomics widely used (16; 17). As the needs have changed, new techniques for transcriptome studies targeting low cell numbers and even more accurately targeted sequencing have emerged (18). In disease research, transcriptome technology can help researchers more accurately understand the pathogenesis of diseases and the relationship between specific RNA and diseases. Based on clarifying the precise regulation of various genes in diseases, transcriptome technology can help in the development of new drugs and has important applications in the prevention and treatment of tumors. The integration and analysis of biological data by various bioinformatics tools are important means of life science research. For example, a network algorithm or Random Forest was used to predict patient-related biomarkers (19). Transcriptome data combined with dual disease analysis can be used to better understand the pathological molecular mechanisms between diseases and make more accurate drug predictions (20). The present study aimed to identify hub genes and a hot research topic to the link between NAFLD and Sarcopenia. Therefore, by obtaining transcriptome sequencing data from clinical patients of the two diseases from the GEO database, further joint analysis of their gene expression data was conducted. The differences and commonalities were preliminarily analyzed to clarify the disease characteristics of NAFLD and Sarcopenia. After that, the co-expressed genes of the two diseases were screened. Diversity statistical analysis methods were used to obtain the co-expressed genes and the pathways significantly associated with NAFLD and Sarcopenia. Finally, we integrate the results from the single analysis and intend to provide a basis for subsequent clinical-related research.

## 2 Materials and methods

### 2.1 Data processing

**Figure 1:**
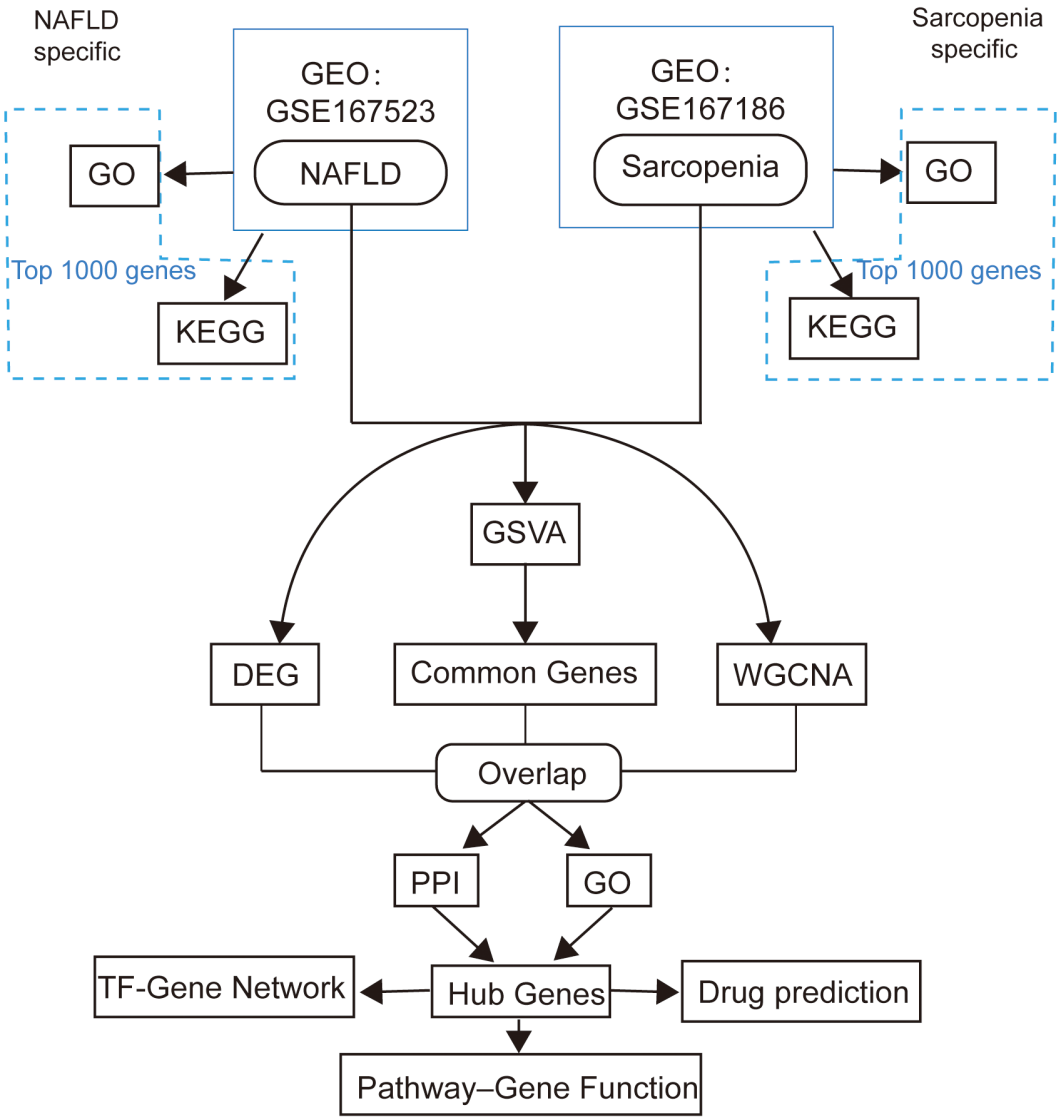
The framework of this study.

Two genome-wide transcriptome profiling using RNA-Seq (GSE167523, GSE167186) of NAFLD and sarcopenia samples were obtained from the GEO database by using Illumina high throughput sequencing platform. 98 NAFLD patients’ gene expression profiles formed the GSE167523 data set. The 72 samples in the GSE167186 data set were patients with sarcopenia. The analytic workflow is shown in **Figure 1**.

### 2.2 Top 1000 expressed genes selection and gene set variation analysis

Counts in NAFLD and Sarcopenia data sets (GSE167523, GSE167186) were normalized treatment. First, the two data sets were integrated according to gene name. Using the cpm function of package R edgeR (V.3.38.4), Counts per million (CPM) were calculated, and log2 was performed. After the log2 (cpm+1) value is arranged from the largest to the smallest, the first 1000 genes are selected as the top 1000 genes. These genes were analyzed by GSVA (Gene set variation analysis) using R package GSVA (V.1.44.5), The reference gene sets were selected from Homo sapiens C5 (ontology gene sets) in the MSigDB database. Use the GSVA function in the GSVA package. The analysis parameters are method=“gsva”, kcdf=“Gaussian”.

### 2.3 Analysis of inter-sample correlation and differentially expressed genes

When analyzing the correlation between samples, the vst function in package R DEseq2 (V.1.36.0) was first used to standardize the expression matrix. Then dist function was used to calculate the Pearson distance between samples, and the prcomp function was used for Principal Component Analysis (PCA). The DESeq function in using DEseq2 gene counts matrix analysis of differentially expressed genes, DEGs judgment standard for pvalue < 0.05 & (log2FoldChange > = 2 | log2FoldChange < = 2), The common genes are pvalue < 0.05 & (log2FoldChange >= -2 & log2FoldChange <= 2). For volcano mapping, the R package EnhancedVolcano (V.1.14.0) is used.

### 2.4 Weighted correlation network analysis

The log2 (cpm+1) matrix with an input file as the gene was constructed using an R package called “WGCNA”. The power value was determined by the pickSoftThreshold function. Weight coexpression network uses the blockwiseModules function. The plotDendroAndColors function draws the clustering between samples. The labeledHeatmap function shows the correlation between the disease and gene Modules. The plotEigengeneNetworks function shows the correlation between each gene Module.

### 2.5 Gene Ontology and pathway enrichment analyses

GO is a database established by the Gene Ontology Consortium that provides simple annotations of gene products in terms of function, the biological pathways involved, and their location in the cell. The Kyoto Encyclopedia of Genes and Genomes (KEGG) pathway is a database dedicated to storing information about genetic pathways in different species. KEGG’s Orthopedic Annotated System (KOBAS) (http://kobas.cbi.pku.edu.cn) is a gene/protein functional annotation and functional enrichment Web server developed by Peking University, which collected functional annotation information of 4325 species. “GO terms” and “KEGG pathways” analyses use the “enrichGO” function and “enrichKEGG” function in the R package clusterProfiler (V.4.4.4), respectively, with the p-value cutoff set to 0.05. GO terms-genes network mapping uses the cnetplot function. The R packages GOplot (V.1.0.2) and ggplot2(V.3.3.6) are also used for visualization.

### 2.6 Determination and functional analysis of hub gene

Search Tool for the Retrieval of Interacting Genes (STRING; http://string-db.org )(11.0 version) Relationships between proteins of interest can be searched, such as direct binding relationships or co-existence of upstream and downstream regulatory pathways, to construct PPI networks with complex regulatory relationships. The TF-genes network is predicted by NetworkAnalyst software. DSigDB database was used to predict possible small-molecule drugs.

## 3 Results

### 3.1 GO and KEGG pathway analyses of NAFLD and sarcopenia

**Figure 2:**
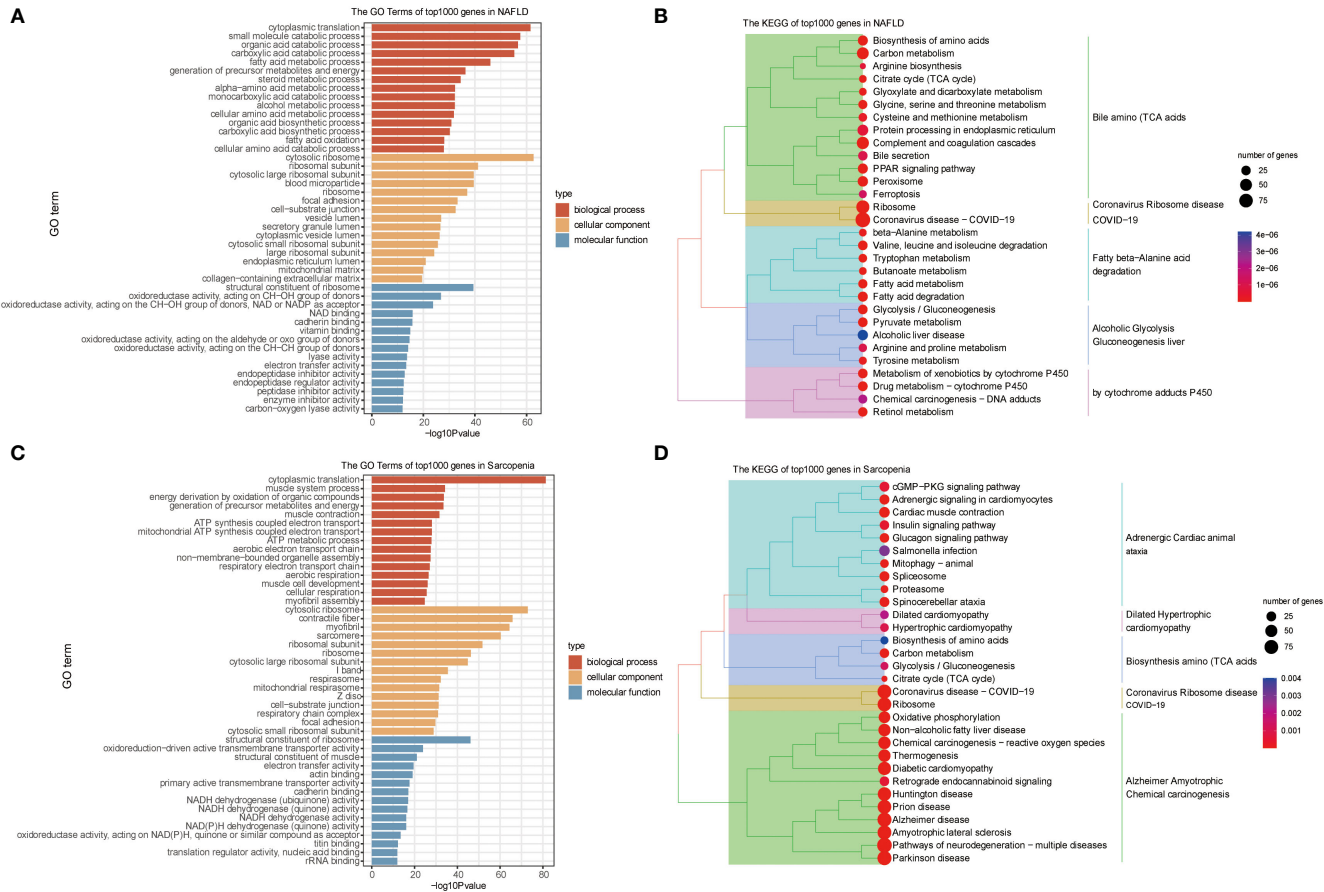
GO and KEGG Pathway Analyses of NAFLD and Sarcopenia. **(A)** The enriched GO terms of the top 1000 genes in NAFLD; **(B)** The KEGG of the top 1000 genes in NAFLD; **(C)** The enriched GO terms of the top 1000 genes in Sarcopenia; **(D)** The KEGG of the top 1000 genes in Sarcopenia.

We conducted a preliminary analytic and statistical study on the disease data from NAFLD and sarcopenia. First, we performed enrichment analysis on the top 1000 genes from 98 NAFLD patients. The observations demonstrate a strong relationship between fat metabolism and energy metabolism in both molecular function, cellular component, and biological process (**Figure 2A**). And when we look at the KEGG data, we can see that these genes are related to some relevant metabolic pathways, such as liver alcoholic glycolysis degradation, fatty beta−alanine acid, cytochrome adducts P450, and other related metabolic pathways shown in **Figure 2B**. Genetic groups related to NAFLD were described.

Then we obtained transcriptome data from 81 patients with sarcopenia and selected the top 1000 genes for the enrichment analysis of GO and KEGG. Sarcopenia genes were significantly correlated with energy metabolism and REDOX pathways in molecular function, cellular components, or biological processes (**Figure 2C**). Dilated Hypertrophic Cardiomyopathy, Biosynthesis of Amino Acids (TCA Acids), and Alzheimer’s Amyotrophic Chemical Carcinogenesis were all strongly associated with KEGG enrichment (**Figure 2D**). This indicates that energy metabolism and redox pathways play a significant role in the disease features of NAFLD and sarcopenia, respectively.

### 3.2 Gene set variation analysis in NAFLD and sarcopenia

**Figure 3:**
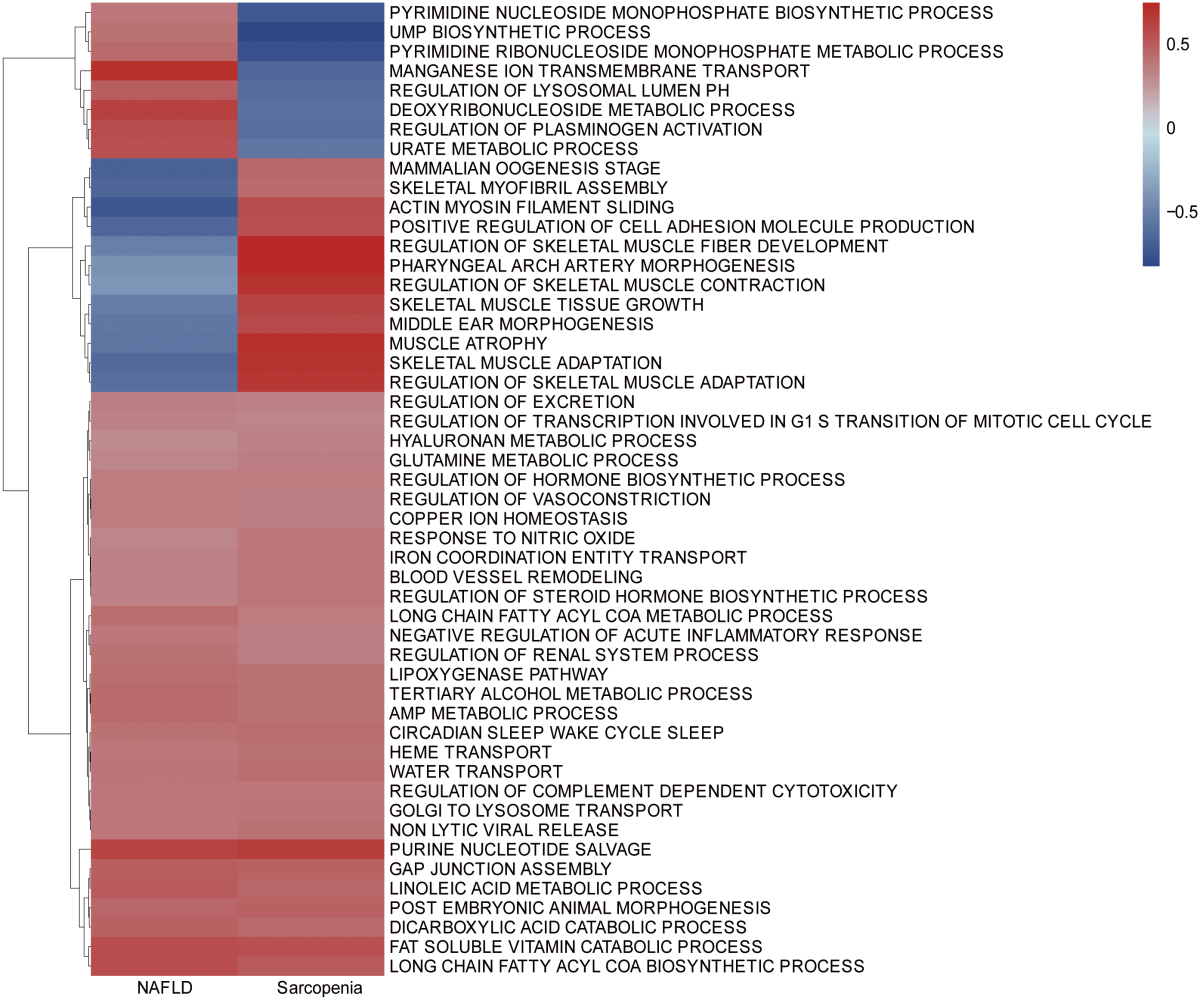
Gene Set Variation Analysis (GSVA) in NAFLD and Sarcopenia. GSVA of top 1000 genes in NAFLD and Sarcopenia.

To further investigate the similarity between the two diseases, we integrated the genes of NAFLD and Sarcopenia. The enriched GSVA of the two diseases showed the main pathways as follows, according to the clustering analysis of the genes expressed in the two diseases: Purine nucleotide salvage, Fat-soluble vitamin catabolic process, Lipoxygenase pathway, Long-chain fatty acyl CoA biosynthetic process, Long-chain fatty acyl CoA metabolic process (**Figure 3**). A high similarity between the two diseases was identified by GSVA.

### 3.3 Differential gene expression in NAFLD and sarcopenia

**Figure 4:**
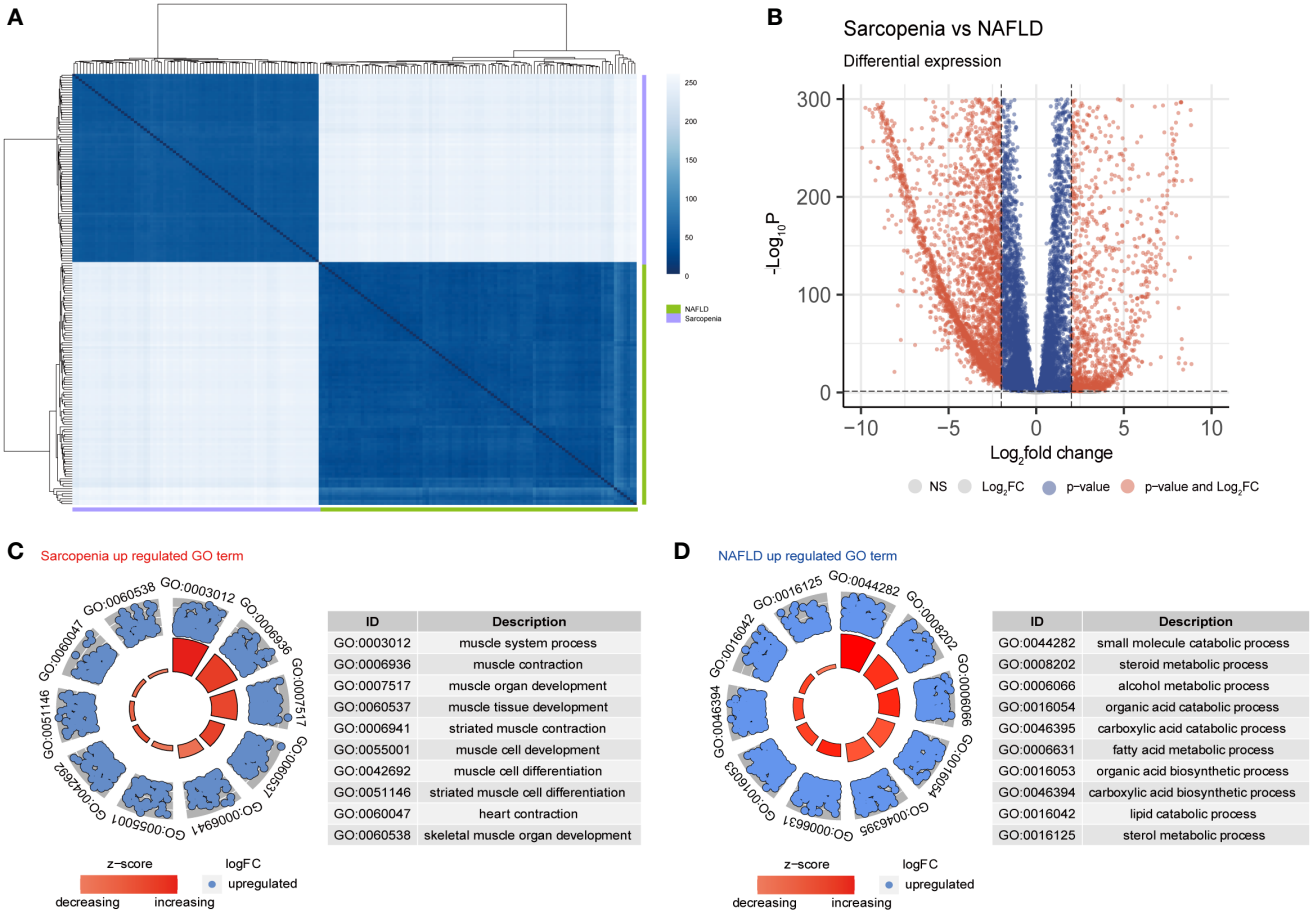
Differential gene expression in NAFLD and Sarcopenia. **(A)** The correlation heatmap of NAFLD and Sarcopenia; **(B)** Volcanic map showing the differentially expressed genes of NAFLD and Sarcopenia; **(C)** The GO term of the up-regulated pathways in sarcopenia versus NAFLD; **(D)** The GO term of the up-regulated pathways in NAFLD versus sarcopenia.

Principal component analysis (PCA), which was used to further evaluate the transcriptome of these two diseases, revealed that the differences between the two diseases were more meaningful than the differences between the two diseases themselves (**sFigure1A**). The Pearson distance analysis, as shown in **Figure 4A**, further supported this result. **Figure 4B** indicates the variations in overall gene expression between the two diseases. The statistics show that there are numerous overlap genes between the two diseases, which will need to be further investigated. Searching at the differences between the two diseases and the differentially expressed genes in sarcopenia versus NAFLD, it is fairly obvious from GO terms that the majority of the genes associated with sarcopenia’s high expression are those that are participated in muscle system processes, muscle contraction, muscle organ development, and other components of muscle development (**Figure 4C**). However, the metabolism of small molecules, sterols, alcohol, and other fat and disease-related metabolic pathways were all strongly expressed by NAFLD (**Figure 4D**). Similar results to those in the GO term were shown in the KEGG enrichment (**sFigure 1C**). Sarcopenia is mostly overexpressed in pathways linked to muscle growth relative to NAFLD; while compared to sarcopenia, NAFLD is overexpressed in lipid metabolism-related pathways.

### 3.4 Common genes analyses in NAFLD and sarcopenia

**Figure 5:**
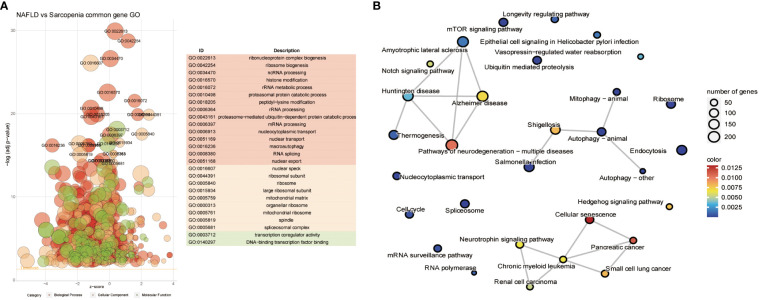
Common Genes Analyses in NAFLD and Sarcopenia. **(A)** The GO term of all common genes in NAFLD and Sarcopenia; **(B)** The KEGG of all common genes in NAFLD and Sarcopenia.

We further investigate the relationship between the two diseases in consideration of the common genes depicted in **Figure 4B**. The resulting Go term revealed the common genes of NAFLD and Sarcopenia, regardless of their molecular function, cellular component, or biological process, by clustering the shared genes. These mainly enriched processes involve ribonucleoprotein complex biogenesis, ribosome biogenesis, ncRNA processing, histone modification, rRNA metabolic process, transcription coregulator activity, On DNA binding transcription factor binding, and other pathways, which indicates that these two diseases are strongly connected to epigenetic changes (**Figure 5A**). After evaluating the KEGG enrichment of common genes, we observed that various relevant pathways were enrichment, as well as metabolic pathways of numerous significant diseases (**Figure 5B**). Two diseases associated with nucleic acid metabolism and epigenetic modifications by GO enrichment.

### 3.5 Weighted correlation network analysisof NAFLD and sarcopenia

**Figure 6:**
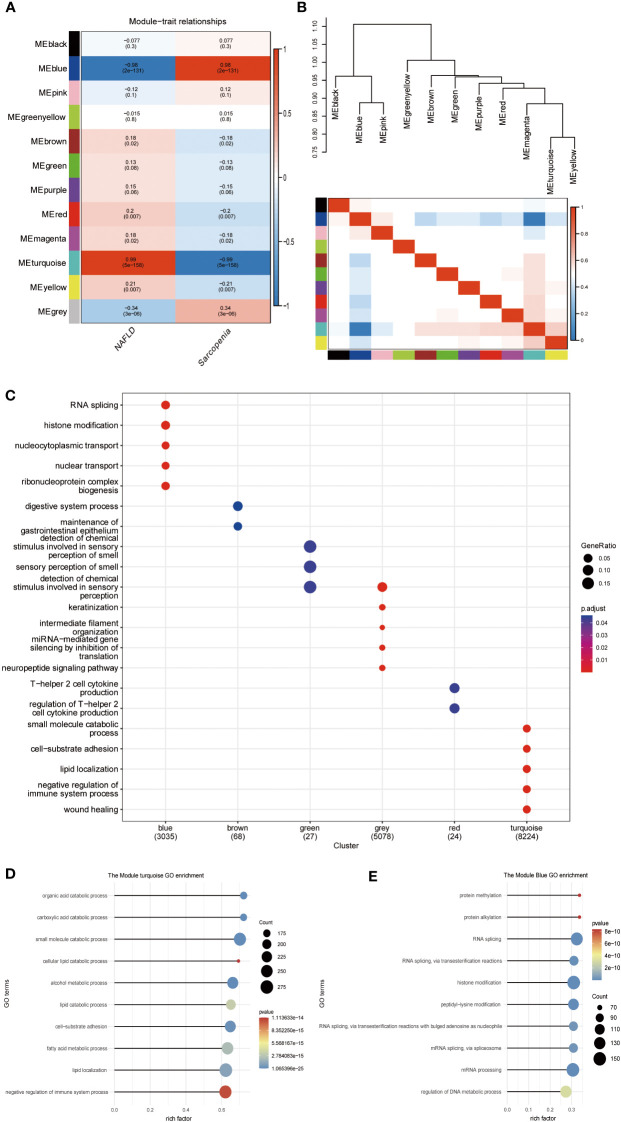
WGCNA of NAFLD and Sarcopenia. **(A)** Module–trait associations. Each row corresponds to a module, and each column corresponds to a trait. Each cell contains the corresponding correlation and P value. The table is color-coded by correlation according to the color legend; **(B)** Eigengene dendrogram and eigengene adjacency plot; **(C)** Gene Ontology analysis; **(D)** Gene Ontology analysis of the genes involved in the turquoise module; **(E)** Gene Ontology analysis of the genes involved in the blue module.

The scale-free network, adjacency matrix, and topological overlap matrix (**sFigure 2A**) were all constructed after the two groups of data were clustered using the Pearson correlation coefficient. Removal of the outliers, a sample clustering tree (**sFigure 2B**) was established. Finally, **Figure 6A** displays 12 modules based on average hierarchical clustering and dynamic tree pruning (the grey module is often regarded as an undefined module). We found that the blue and turquoise modules, which were selected as clinically significant modules for further analysis, were significantly correlated with NAFLD and sarcopenia. We investigated the connection between characterizing genes. Information regarding the pairing relationships between gene co-expression modules can be obtained from characteristic genes. The characteristic genes were grouped. The results demonstrated that the 11 modules can be grouped into two clusters in **Figure 6B** and that each of the module combinations (blue and pink, and turquoise and yellow) exhibit a high level of interactive connectedness. We enriched the modules for GO terms by combining them with clinical characteristics (**Figure 6C**). Blue modules were found to be significantly correlated with histone modification and RNA splicing, whereas brown modules were associated with gastrointestinal diseases, green modules with olfactory dysfunction, gray modules with miRNA regulation, red modules with cofactor 2, and turquoise modules with lipid metabolism. Which, the turquoise module also indicated that the organic acid catabolic process, carboxylic acid catabolic process, small molecule catabolic process, cellular lipid catabolic process, and alcohol metabolic process were significantly correlated (**Figure 6D**). Blue module revealed that protein methylation, protein alkylation, RNA splicing, and RNA splicing *via* transesterification processes were all strongly related to both diseases (**Figure 6E**). WGCNA shows that metabolism-related processes and behaviors such as RNA shearing are closely associated with both diseases.

### 3.6 Protein-protein interaction network

**Figure 7:**
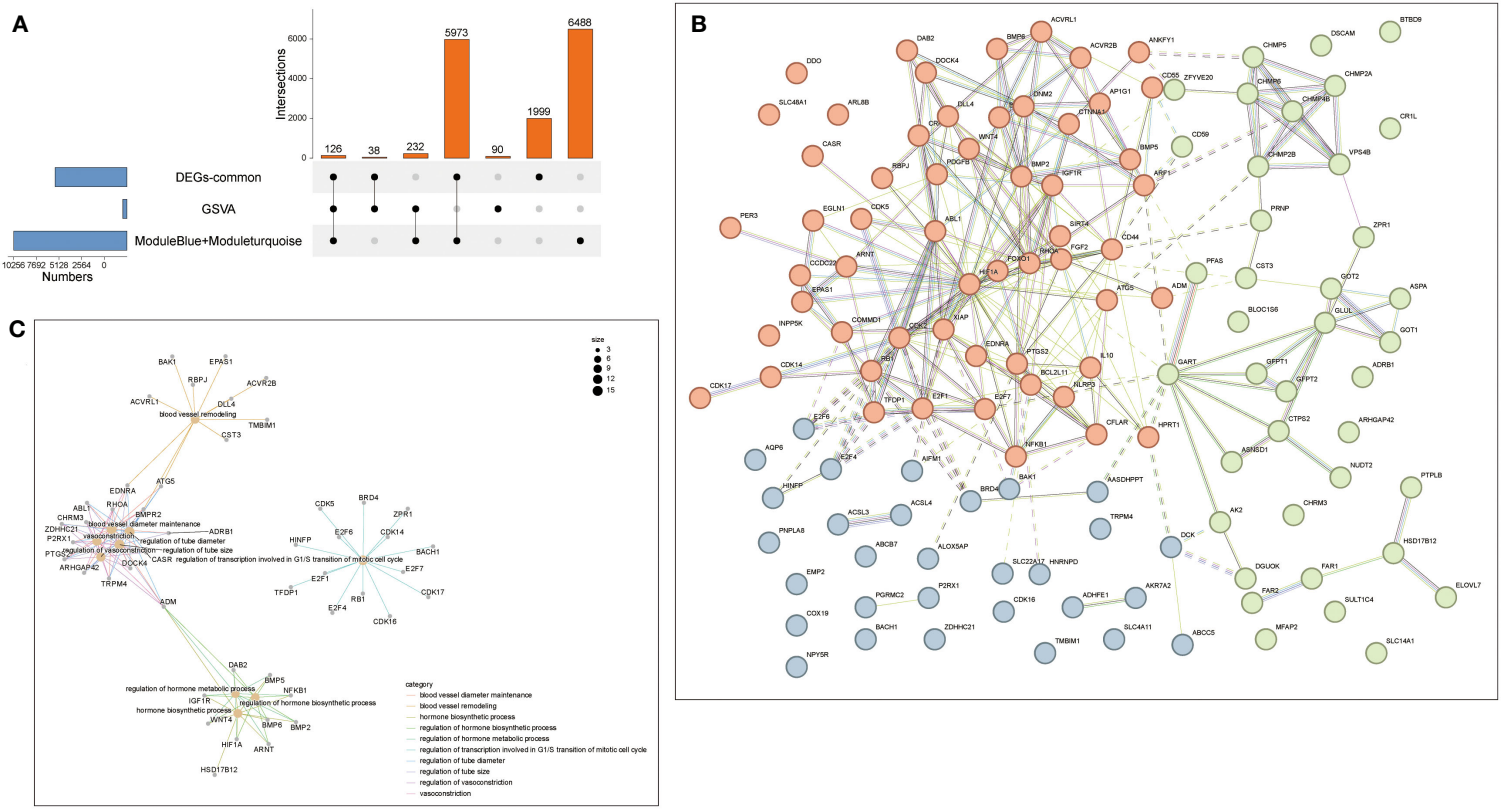
Protein-Protein Interaction Network (PPI). **(A)** The Venn diagram showed that seven algorithms have screened out 16 overlapping hub genes in NAFLD and Sarcopenia. **(B)** PPI network diagram. **(C)** The GO biological process analyses overlap genes from **(A)**.

Therefore, intersection analysis was conducted on the genes in the obtained GSVA, DEG of common genes, and the modules obtained by WGCNA. 126 genes were screened out from these intersection genes for subsequent analysis (**Figure 7A**). Therefore, The PPI network of the intersection DEGs was constructed using String (**Figure 7B**). We analyzed the enrichment top GO pathway by looking at the GO of intersection genes and found that these genes and blood vessel remodeling, regulation of transcription involved in G1/S transition of the mitotic cell cycle, regulation of hormone biosynthetic process, and other vascular regulation and hormone anabolic pathways (**Figure 7C**). Two pairs of genes with high and low expression were filtered out by combining the results of **Figures 7B, C**. The results showed that the PPI network of these 126 genes correlated with energy, and hormone anabolism.

### 3.7 Pathway–gene functional network to screen hub gene

**Figure 8:**
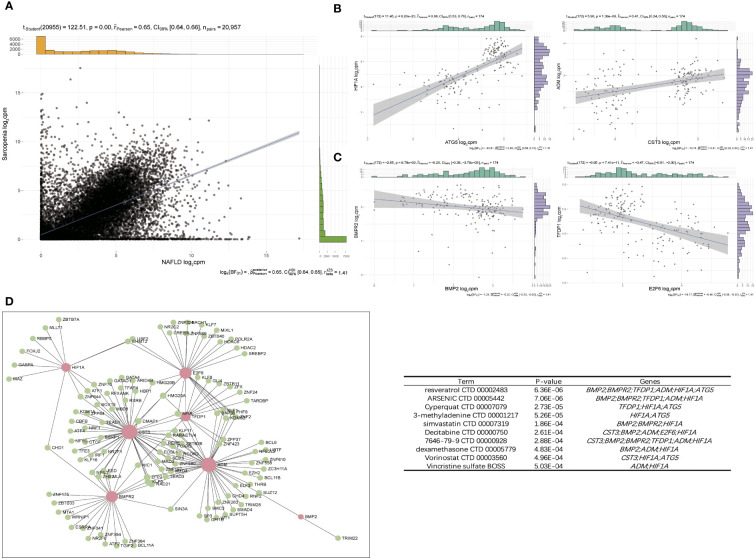
Pathway–Gene Functional Network to screen hub gene. **(A)** Correlation of overlap genes in NAFLD and Sarcopenia. **(B)** Two pairs of genes that were positive-regulated expressed. **(C)** Two pairs of genes that were negative-regulated expressed. **(D)** The TF–target network of periimplantitis. TF, transcription factor.

The expression patterns of NAFLD and Sarcopenia were found to be positively correlated after reviewing the expression values of all genes. This finding implies that the two diseases may be attached by a co-regulatory network and that further research into the co-regulatory mechanisms of the two diseases is essential (**Figure 8A**). **Figure 8B** shows the high expression of *HIF1A* and *ATG5*, as well as *ADM* and *CST3*, in the two diseases. *HIF1A* and *ATG5* were also strongly connected with the reoxidation-reduction of the disease. *ADM* and *CST3* were linked to hormonal disorders. The mutually compatible receptors *BMP2* and *BMPR2*, which are connected to protease hydrolysis, etc., were two pairs of genes with low expression in common. *TFDP1* and *E2F6* were two genes that also have significant regulatory functions in transcription and translation (**Figure 8C**). Additionally, we constructed a TF-target regulatory network diagram based on the eight-node genes, and **Figure 8D** clearly illustrates the correlation between the eight genes. With a high degree of linkage, *CST3*, *TFDP1*, *ADM*, and *BMPR2* could be especially noteworthy in the TF-target network. To give thorough treatment for patients who also have NAFLD and sarcopenia, several additional TFs were included, and medicines prediction was also done based on these genes. The top 10 potential medicines were displayed in **Table 1**. Future treatments for both diseases may be based on these 8 node genes.

**Table 1:** Top 10 drug predictions of hub genes.

Term	Overlap	P-value
resveratrol CTD 00002483	6/1602	6.36E-06
ARSENIC CTD 00005442	5/854	7.06E-06
Cyperquat CTD 00007079	3/160	2.73E-05
3-methyladenine CTD 00001217	2/028	5.26E-05
simvastatin CTD 00007319	3/305	1.86E-04
Decitabine CTD 00000750	5/1801	2.61E-04
7646-79-9 CTD 00000928	6/3095	2.88E-04
dexamethasone CTD 00005779	3/422	4.83E-04
Vorinostat CTD 00003560	3/426	4.96E-04
Vincristine sulfate BOSS	2/86	5.03E-04

## 4 Discussion

Investigating embryonic development, we learned that mesodermal differentiation is the principal source of muscle formation (21), while the liver consists of endoderm-derived hepatobiliary cell lineage and various mesodermal-derived cells (22), and liver development, from liver specification to liver maturation, requires close interaction with cells of mesodermal origin. This also implies that the progenitor cells of both organs have a strong commonality, and the origin from the same germ layer indicates that they are also functionally very closely related.

To better identify genes that are co-regulated in both diseases, we used the GSVA, common gene in DEGs analysis, and WGCNA analyses to jointly identify genes that are expressed in high abundance in both diseases and involved in disease development. Our research focused on the analysis of the co-expressed genes in both tissues to identify potential therapeutic strategies. We conducted an enrichment analysis for the top 1000 genes associated with each disease after homogenizing the obtained data. While Sarcopenia was more closely associated with energy metabolism and reoxidation reduction, we could see that the principal genes expressed in NAFLD were still associated with lipid metabolism, glycometabolism, and energy metabolism (**Figure 2**). As the body’s primary metabolic organs, the liver and muscle can also be considered as potentially sharing some of the same functions (23). After realizing this commonality, we investigated the variations and consistency of the data in more detail. We proceeded by analyzing the differences between the two diseases and the co-expressed genes. We could see that sarcopenia and NAFLD were distinguishable in that sarcopenia had a higher expression of genes mainly related to muscle development. The high gene expression of NAFLD relative to sarcopenia was enriched in the adipose metabolism pathways associated with NAFLD illness itself (**Figure 4E**), which also reflected the specificities of each disease. This was associated with the gene expression of the muscle itself (**Figure 4D**). We were particularly interested in the relationship between the two diseases in our research. The major pathways of co-expressed enrichment of these two diseases were identified by GSVA, and the results revealed that the enrichment pathway was not only significantly related to epigenetics but also involved in other metabolic diseases and immune-related pathways (**Figure 3**). This suggests that the gene expression of most metabolic diseases is very similar, and the cause of metabolic diseases may be related to REDOX-related pathways (24), epigenetic modification, or the mutual regulation of various RNAs (25, 26), as well as activating the immune system (27, 28).

WGCNA was used for further analysis to check the relationship between these two diseases in more depth. In the highly correlated turquoise and blue modules, we found that the key genes for the two diseases were still enriched in the epigenetic modification and lipid metabolism pathways (**Figure 6D, E** **)**. Additionally, it is consistent with the preliminary results. In addition to checking more relevant genes and more precisely identifying and verifying the core genes of the two diseases, we chose the intersection of genes obtained by various analysis methods. These 126 genes were shown to be significantly enriched for the cell cycle, angiogenesis, and hormone anabolism pathways. We further checked into the co-expression of these genes and observed 4 pairs of genes out of a large number that were concurrently positive- or negative-regulated. *HIF-1A* activates the transcription of numerous genes, including those involved in energy metabolism, angiogenesis, apoptosis, and other genes whose protein products increase oxygen delivery or facilitate metabolic adaptation to hypoxia, serving as a master regulator of cellular and systemic homeostatic response to hypoxia (29); *ATG5* encoded protein participates in several cellular functions, including the production of autophagic vesicles, mitochondrial quality control following oxidative damage, inhibition of the innate antiviral immune response, and proliferation and development of lymphocytes (30). We also observed that the preprohormone *ADM*, which is produced by this gene, can be broken down into two physiologically active peptides: adrenomedullin and pro-adrenomedullin N-terminal 20 peptide. Adrenomedullin is a 52 AA peptide having a variety of activities, such as vasodilation, hormone secretion regulation, angiogenesis stimulation, and antibacterial action. It also plays a significant role in oxidative stress (31); *CST3* inhibitors appear to have preventive properties in a variety of human fluids and secretions, but they also play a crucial regulatory role in the development of cancer and other diseases (32) (**Figure 8B**). These genes have a strong connection to the REDOX of the disease. Therefore, synergistic high expression in NAFLD and sarcopenia is significant.

However, there are fewer studies related to the direct occurrence of RNA splicing in NAFLD, but lipid accumulation, as well as obesity, are closely associated with the development of NAFLD. Which is the main cause of increased alternative RNA splicing in the liver. Gene expression data from the liver and muscle of Pihlajamaki et al. provided that obese patients found substantial downregulation of RNA splicing genes, suggesting that the expression of RNA splicing-related genes is negatively associated with liver lipids accumulation and hyperinsulinemia and that altered expression of RNA splicing factors may contribute to obesity-related phenotypes (33). Also, NAFLD, especially sarcopenia, as a disease of the elderly, is significantly associated with increased RNA splicing (34). For example, Li et al. published an article in Cell Metabolism demonstrating that death-associated protein kinase-related apoptosis-inducing kinase-2 (*DRAK2*) can inhibit the phosphorylation of *SRSF6* by the *SRSF* kinase *SRPK1*, and regulates selective splicing of mitochondrial function-related genes (35).

The activation of BMP signaling in skeletal muscle is significant in maintaining muscle mass as well as muscle-nerve interaction during cachexia and the aging process (36, 37). Restoring *BMP* activity ameliorates cancer-mediated muscle wasting and sarcopenia (36, 37). The activity of *BMP* receptors in muscles induced hypertrophy was dependent on Smad1/5-mediated activation of mTOR signaling (38). *TFDP-1* is a heterodimerization partner for members of the E2F family of transcription factors and up-regulates *E2F*-mediated transcriptional activation (39). *E2F/TFDP-1* regulates the expression of various cellular promoters, particularly gene products that are involved in the cell cycle (40). The combination of *TFDP1* with *E2F*s can promote liver regeneration by regulating *MYCN* transcription (41). Elevated expression of *TFDP1* was associated significantly with larger tumor size and down-regulation of *TFDP1* inhibited the growth of Hep3B cells. In conclusion, overexpression of *TFDP1* may contribute to the progression of some HCCs by promoting the growth of the tumor cells (40). Murine and human HCC data indicate significant correlations of *STMN1* expression with *E2F1/TFPD1* and with *KPNA2* expression and their association with poor prognosis in HCC patients (42). These four genes are negatively regulated and there are opposite regulatory patterns, and we checked their roles and found that the mechanisms are also different in the two diseases.

We also examined the TF regulatory network for these 8 genes, and we found that several of the transcription factors among these genes had strong connections to fibrosis, damage, and fat metabolism (**Figure 8D**). Resveratrol, which ranked top among such genes to predict small molecule medicines, was discovered to have a beneficial preventative impact on obesity-induced diet in NAFLD and NASH patients (43) (**Table 1**). It can also improve the validation status of skeletal muscles (44). Resveratrol is also a highly significant healthcare product in daily life, demonstrating the necessity of a daily supplement. In addition, the last few medicines are also widely used. This evidence can support the continued usage of previously prescribed medicines.

When compared to other research, our study still has several limitations. For example, disease development may be regulated at various histological levels, and we have only conducted a preliminary investigation of the co-regulatory mechanisms of NAFLD and sarcopenia at the transcriptome level. For instance, studies on DNA/RNA methylation have been applied to explain how so many diseases develop (39). Our analysis also revealed a strong correlation between both diseases and lipid metabolism as well as oxidative stress, demonstrating the importance of further metabolomic research (26, 45). What’s more, the research should really be based on healthy samples to obtain differentially expressed genes and then compare them. However, since healthy human liver and muscle samples are not easy to obtain, we only collected partial liver control datasets, but considering that the liver’s gene expression is affected by sex and age (46) (47), we were unable to find a dataset that could be matched exactly. The dataset was not available for muscle. Therefore, the study was mainly conducted on the expression profile of the disease. Therefore, it is necessary to update the data in healthy subjects if they are available subsequently.

In summary, our work demonstrates the potential transcriptome regulatory mechanisms of NAFLD and sarcopenia. Through a thorough mapping of the transcription pattern, the key genes, molecular processes, and potential therapeutic targets that cause NAFLD and sarcopenia were examined. It offers a new perspective and supporting evidence to decrease the high incidence of NAFLD in sarcopenia patients.

## Data availability statement

Publicly available datasets were analyzed in this study. This data can be found here: The datasets presented in this study can be found in online repositories. The names of the repository/repositories and accession number(s) can be listed below. Repository/Repositories Accession Number Gene Expression Omnibus GSE167523 Gene Expression Omnibus GSE167186.

## Author contributions

JY and FY designed the study. ZX and ZY performed data analysis. ZX prepared the figures and tables. ZX, ZY, and SL wrote the manuscript and approved the final draft. ZX, ZY, and ZT participated in data interpretation and analysis. ZX, ZY, and SL were involved in proofreading and deep editing and approved the final manuscript. JY and FY devised the main conceptual idea, supervised the project, performed proofreading and deep editing of the manuscript, and approved the final draft. All authors contributed to the article and approved the submitted version.

## Supplementary material

Supplementary Figure 1 Differential gene expression in NAFLD and Sarcopenia. (A) The PCA gene expression profile in NAFLD and Sarcopenia patients; (B) The KEGG enrichment of the up-regulated pathway in sarcopenia; (C) The KEGG enrichment of the up-regulated pathway in NAFLD.

Supplementary Figure 2 Determination of soft-threshold power in the WGCNA. (A) Analysis of the scale-free index for various soft-threshold powers (β). (B) Analysis of the mean connectivity for various soft-threshold powers.
