# Supplementary figures and images for: Exploration of the core gene signatures and mechanisms between NAFLD and sarcopenia through transcriptomic level
Source: Front Endocrinol (Lausanne). 2023 Mar 9;14:1140804. doi: 10.3389/fendo.2023.1140804 (PMC10033966; doi:10.3389/fendo.2023.1140804)

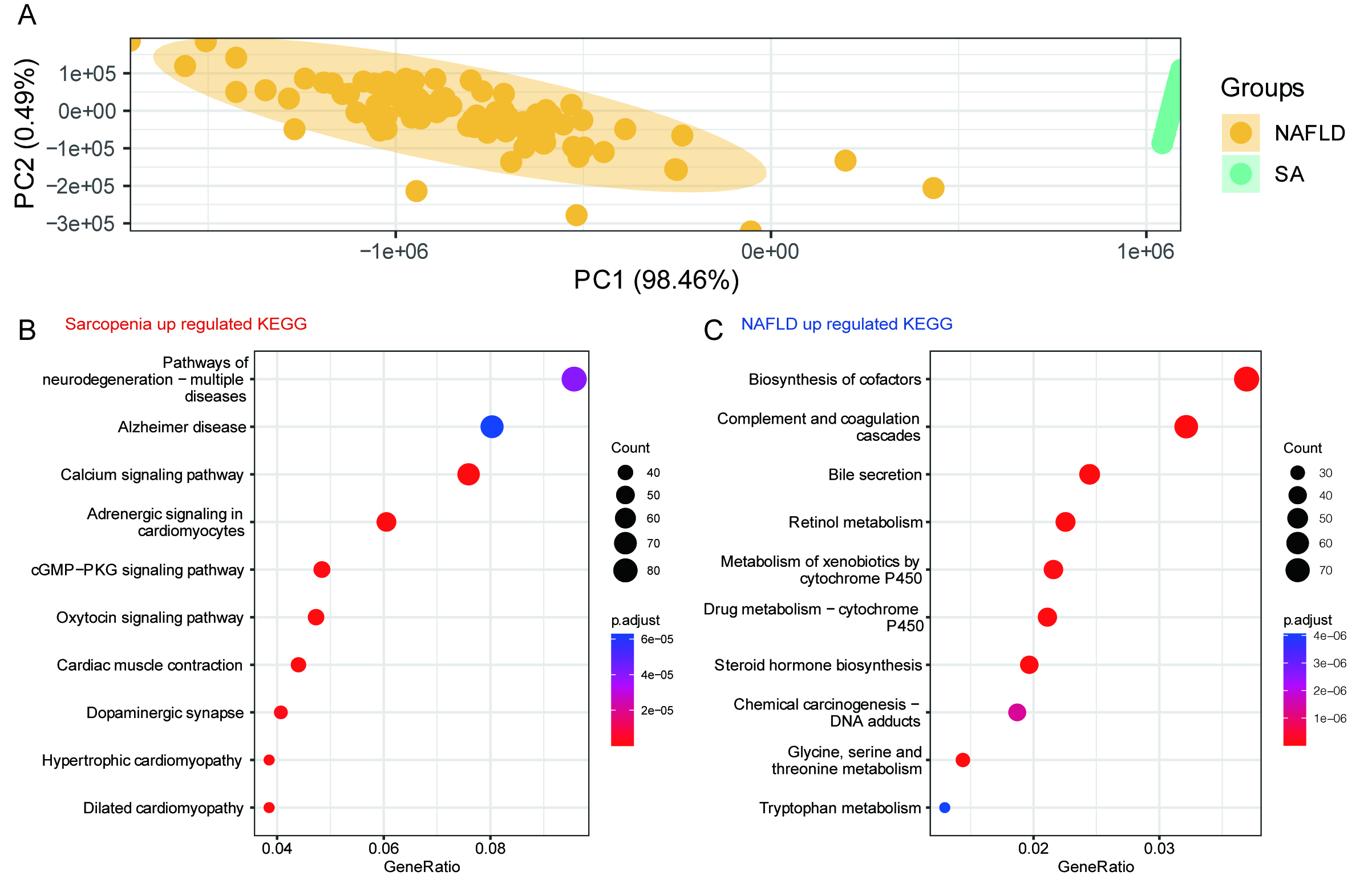

Supplement: Supplementary Figure 1 — Differential gene expression in NAFLD and Sarcopenia. (A) The PCA gene expression profile in NAFLD and Sarcopenia patients; (B) The KEGG enrichment of the up-regulated pathway in sarcopenia; (C) The KEGG enrichment of the up-regulated pathway in NAFLD. [file Image_1.tif]

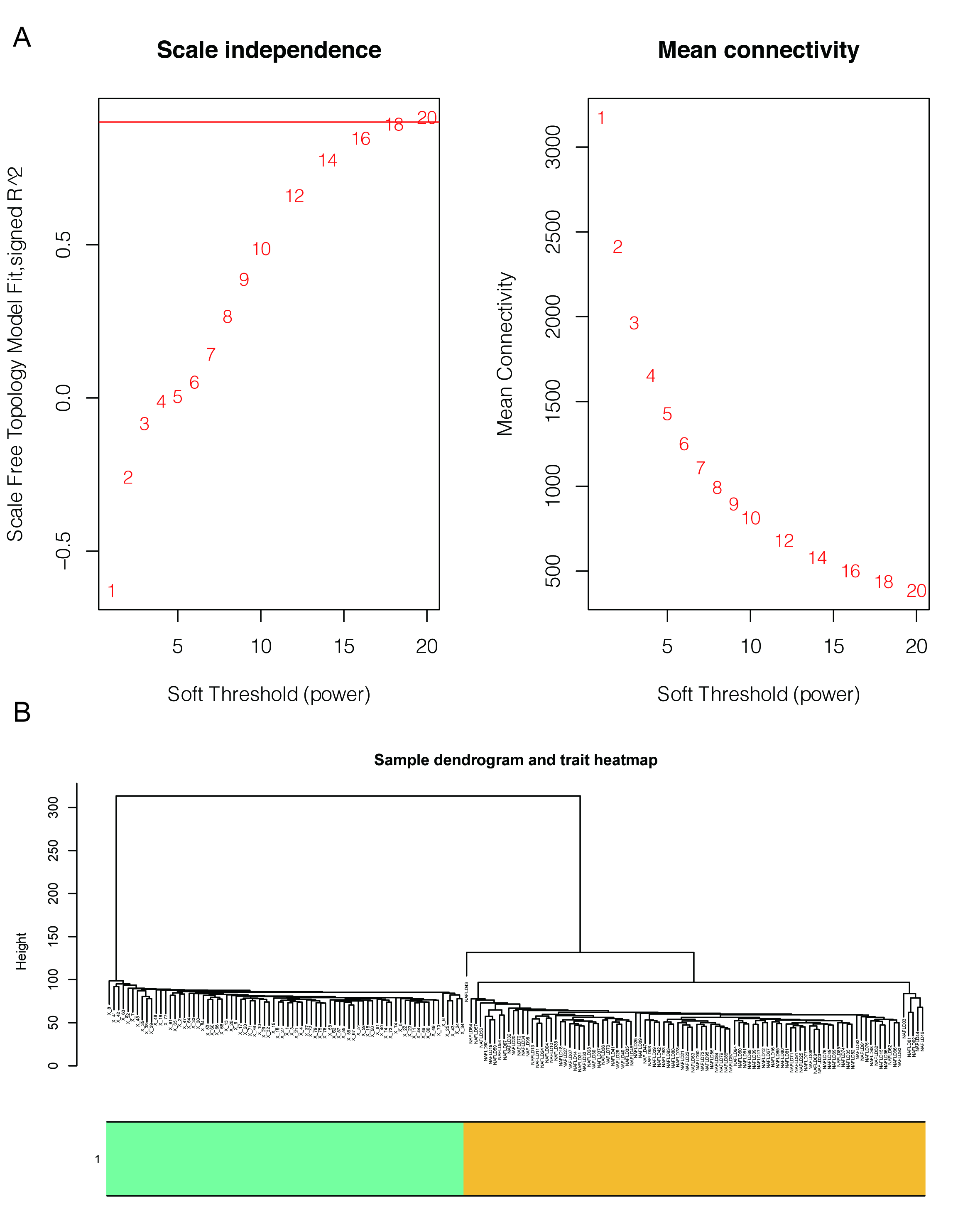

Supplement: Supplementary Figure 2 — Determination of soft-threshold power in the WGCNA. (A) Analysis of the scale-free index for various soft-threshold powers (β). (B) Analysis of the mean connectivity for various soft-threshold powers. [file Image_2.tif]
